# Phylogenetic and Functional Analysis of β-Glucanases in Tannerella forsythia Reveals Distinct GH16 Family Glycoside Hydrolase Lineages in Oral Bacteria

**DOI:** 10.21203/rs.3.rs-9916188/v1

**Published:** 2026-06-25

**Authors:** Sreedevi Chinthamani, Rajendra P. Settem, Kathryn M. Kauffman, Ashu Sharma

**Affiliations:** University at Buffalo; University at Buffalo; University at Buffalo; University at Buffalo

## Abstract

*Tannerella forsythia* is an asaccharolytic anaerobic bacterium whose presence in oral biofilms is associated with poorer outcomes for periodontitis. *T. forsythia* expresses a β-glucanase enzyme, TfGlcA, that is upregulated on contact with the pathobiont *Fusobacterium nucleatum*, supporting the latter’s growth through release of glucose oligomers. Here, we systematically characterize the enzymatic properties of TfGlcA and investigate the extent to which its genomic architecture is common among oral β-glucanases (also in Glycoside Hydrolase Family 16 Subfamily 3, GH16_3). First, we evaluated the substrate specificity, kinetics, and structural features of TfGlcA. TfGlcA displayed highest activity toward laminarin, followed by lichenin, with minimal activity toward yeast β-glucan, and no detectable activity against chitin or carboxymethyl cellulose. These findings on proxy-substrates indicate strong preference for β1,3-linked glucose polysaccharides, enriched in dietary plant cell walls. Second, we investigated the phylogenetic diversity and genome-neighborhoods of oral GH16_3s. GH16_3 genes were found in 15% of oral bacterial species, representing phylogenetically diverse taxa. A subset of genes, including TfGlcA, occur in polysaccharide utilization loci (PUL), with the TfGlcA PUL overall unique in its association with an adjacent extracytoplasmic function sigma/anti-sigma factor regulatory module. A second GH16_3 in T. forsythia, GlcB, falls within a large clade of sequences dominated by Bacteroidota and lacking PUL architecture. This work represents the first systematic characterization of substrate specificity and kinetics of an oral β(1,3) glucanase and suggests that *T. forsythia* plays a distinctive role in shaping public-goods nutrient dynamics in the periodontal niche.

## Introduction

*T. forsythia* is implicated in the pathogenesis of periodontitis^1–3^, a chronic inflammatory disease that leads to alveolar bone loss and is a potential risk factor for systemic conditions including cardiovascular/atherosclerosis, diabetes, rheumatoid arthritis, and low weight preterm births. *T. forsythia* is frequently present in all stages of periodontitis^4^. The baseline *T. forsythia* levels are associated with poorer treatment outcomes for periodontitis^5^. Machine learning algorithms predict *T. forsythia* as a significant microbial species distinguishing periodontitis from subgingival health, highlighting its potential role in dysbiosis-associated disease progression^6^. In addition to periodontitis, it is associated with endodontic and dental implant infections^7–10^.

We have previously shown that *T. forsythia* produces a GH16 family β-glucanase enzyme, TfGlcA, whose expression is significantly upregulated in response to *Fusobacterium nucleatum*, an oral bridge bacterium with which *T. forsythia* forms synergistic biofilms^11^ and coexists in a close physical proximity in the subgingival biofilms^12–14^. The expression of TfGlcA is regulated by an extracytoplasmic function (ECF) sigma/anti-sigma regulatory system^15,16^. In addition to TfGlcA, the *T. forsythia* genome codes for a second GH16 gene, GlcB, which has not been characterized. GH16 glycosyl hydrolases are group of enzymes that differentially act on β (1,4) or β(1,3) glycosidic bonds in glucan substrates to release glucose oligosaccharides. It has been shown that in co-cultures of *T. forsythia* and *F. nucleatum*, TfGlcA mediated release of glucose oligosaccharides as a carbon source from lichenin β-glucan, thereby promoting *F. nucleatum* growth. Different types of β-glucan polysaccharides are potentially available from the diet - e,g., mixed-linkage β (1,3/1,4) glucans (MLGs) in oats and barley, β (1/3) glucan (callose) in plant cell walls, and branched β (1,3)(1,6) glucans in edible mushrooms and seaweeds^17,18^. Additionally, *Candida albicans*, a commensal fungal organism of the oral microbiome, represents an additional potential source of β-glucan substrate for *T. forsythia*. In this regard, there is strong evidence that *C. albicans* releases and exposes β−1,3-glucan in the oral environment, particularly within biofilm matrices^19,20^. Therefore, it is likely that collective breakdown of different β-glucan sources by TfGlcA might be critical in enhancing the growth of select bacteria utilizing glucose as an energy source.

This study was undertaken to systematically evaluate the substrate specificity, kinetic parameters, and structural features of GH16 β-glucanase genes TfGlcA and GlcB in *T. forsythia*. TfGlcA has been previously shown to hydrolyze lichenin^15,16^, but its full substrate spectrum and enzymatic properties remained unclear. Here, we purify recombinant TfGlcA from *E. coli* and present a detailed analysis of its substrate specificity and structural characteristics. This is the first report that describes the function activity of a β(1,3) glucanase enzyme expressed by an oral bacterial species. We also conducted a comparative phylogenetic analysis of GH16 hydrolases across oral bacteria, which showed that TfGlcA and GlcB are sequence-diverse and represent distinct genomic architectures, with TfGlcA unique in occurring in a polysaccharide is conserved with an adjacent sigma/anti-sigma factor regulatory module.

## Materials and Methods

### Cloning, expression and purification of recombinant TfGlcA

DNA fragments corresponding to the *glcA* (TPE17275.1) and *glcB* (TPE18055.1) open reading frames (ORFs) were codon-optimized for expression in *E. coli* (codon optimized sequences included as supplemental data, Figure S1). These were then synthetically produced, and each subcloned into a pUC-derived vector by GenScript Inc. (Piscataway, NJ). To generate the expression plasmids, DNA fragments encoding the *T. forsythia* β-glucanase GlcA and GlcB proteins were PCR-amplified from the corresponding constructs as templates using Q5 high-fidelity polymerase (NEB). For GlcA, the primers used were: forward primer 5′-GGGGATCCAACAAACAGAAAGAAGAAAAACGTCCGAATGAA-3′ and reverse primer 5′-ATGAAT TCTTAATGGTGATGGTGATGGTGGCTTGAGGAGACCGAGTTCGCTTGTT-3′. For GlcB, the primers used were: forward primer 5′-GGGGATCCGATCAGAAACAAGACGCTTGGC-3, and reverse primer 5′-GAATTCTTAGTGGTGGTGGTGGTGGTGCTTTTGCTTCTGGTAGAAACGAACCCAAT-3′. The primers were designed to exclude signal peptides and lipidation cysteines predicted by PSORTb software^21^ and included *Bam*H1/*Eco*R1restriction sites to the 5’ and 3’ ends respectively, and a 6x His coding sequence to the 3’ end of the amplified DNA product. The amplified fragment and expression plasmid pGEX-2T were each digested with *Bam*HI and *Eco*RI enzymes and ligated together. After transformation into *E. coli* DH5α, positive transformants were screened by PCR and verified by DNA sequencing. Recombinant plasmids containing the correct in-frame inserts for GlcA and GlcB were selected and designated pGEX-GlcA6His and pGEX-GlcB6His, respectively. Each plasmid was then transformed into *Escherichia coli* BL21 (Thermo Fisher Scientific) competent cells for protein expression

### Protein expression and purification

For recombinant protein production, *E. coli* BL21 cells harboring the expression vector pGEX- GlcA6xHis was inoculated into LB medium supplemented with ampicillin (100 μg/ml) and grown overnight at 37°C. The overnight culture was inoculated (1:50 dilution) into a fresh 2YT broth (tryptone 16 g/l, yeast extract10 g/L and NaCl 5 g/L) containing 100 μg/ml ampicillin and grown at 37°C to an OD600 of 0.6, induced with 0.1 mM IPTG and further cultivated for 18 h at 18°C. The culture was centrifuged at 12,000 g for 10 min at 4°C. Cells were washed and suspended in TBS (50 mM Tris-HCl, 150 mM NaCl, pH 7.2) buffer, lysed by sonication on ice. This was followed by addition of Triton X-100 to the lysate to a final concentration of 1% and incubation at 4°C for 15 min with gentle rocking. The protein lysate was then centrifuged at 17,000 g for 15 min at 4°C to remove the cell debris and the supernatant was applied to a nickel affinity chromatography column (His-Trap, 1 mL; GE Healthcare) preequilibrated with the binding buffer (TBS containing 1% Triton x-100). After washing the column with 15 mL of the wash buffer (TBS containing 20 mM imidazole), the protein was eluted with 5 mL of the elution buffer (TBS with 250 mM imidazole). The eluted fraction was extensively dialyzed against TBS at 4°C, and protein concentrations was determined by Coomassie dye binding assay (Pierce). The GST-TfGlcA fusion protein was digested with Thrombin (5 U per mg protein) at 40°C overnight and the thrombin was removed from the digest using a benzamidine affinity column (GE Healthcare) as per the manufacturer’s recommendations. Finally, the cleaved recombinant TfGlcA protein with 6xHis tag was purified by nickel affinity chromatography as above. The purity of the recombinant TfGlcA (rTfGlcA) protein was determined by sodium dodecyl sulfate-polyacrylamide gel electrophoresis (SDS-PAGE) and lichenin zymography as described by our group previously^15^.

### Substrate specificity assay

The substrate specificity of TfGlcA was assayed against 0.2% of each substrate: lichenin (Millipore Sigma, Burlington MA), laminarin (Millipore Sigma), yeast β-glucan (alkali soluble from Megazyme, Wicklow, Ireland), chitin and carboxymethyl cellulose (CMC; MilliporeSigma). The reaction mixture containing the insoluble polysaccharide (yeast β-glucan) was oscillated during incubation. After incubation the reaction mixture was centrifuged at 12, 000 g for 5 min and the supernatant was assayed using 3,5-dinitrosalicylic acid (DNSA; MilliporeSigma, Burlington MA) method^22^.

### Temperature and pH optima determination

To determine the optimum reaction temperature of TfGlcA, reaction temperatures 20, 25, 30, 35, 40, 45, 50, 55, 60, and 65°C were selected. For determination of the optimum reaction pH of TfGlcA, 0.2% laminarin solutions different pH levels were prepared in a citric acid-Na_2_HPO_4_ buffer (pH 3.0–5.0), Na_2_HPO_4_-NaH_2_PO_4_ buffer (pH 6.0–8.0), and a Na_2_CO_3_-NaHCO_3_ buffer (pH 9.0–10.0). The enzyme activity of each group at the optimum temperature was then determined as described above.

### β (1,3)-glucanase activity assay

The β(1,3)-glucanase activity was carried out by measuring the amount of reducing sugars released from a β−1,3-glucan substrate (laminarin) measured by the 3,5-dinitrosalicylic acid (DNSA) method^22^. For a typical assay, 25 μl of laminarin solution (2 mg/mL) was incubated with 25 μl of enzyme solution (75 μg/ml) in 20 mM sodium acetate buffer (pH 6.4) at 40°C for 20 min. After incubation, 100 μl of DNSA reagent was added, mixture boiled for 5 min and absorbance was read at 540 nm after cooling. The content of reducing sugar was determined by generating standard curve of glucose concentration. For estimation of enzyme activity, one enzyme unit (U) was defined as the amount of enzyme required to release 1 μmol of reducing sugar (glucose equivalent) per minute under the experimental conditions. The assay was performed with laminarin under the optimal conditions. For the enzyme kinetic assay, 0.5–20 mg/mL laminarin substrate concentrations were used to determine enzyme velocities. The *K*_*m*_ and *V*_*max*_ values were obtained from Lineweaver-Burk plot (Prism Software).

### Phylogenetic diversity and genome neighborhood analyses

To understand the phylogenetic diversity and genomic contexts of GH16 subfamily 3 (GH16_3) β -glucanases encoded by oral microbes, we identified and analyzed candidate GH16_3 genes in the Human Oral Microbiome Database^23,24^ (HOMD, https://www.homd.org/), as follows.

All HOMD genomes (8,177 genomes, V11.02, downloaded to the UB Center for Computational Research compute cluster (http://hdl.handle.net/10477/79221) on October 30, 2025) were annotated using standalone run_dbcan v5.2.8^25^ from input genome sequence fna files. All run_dbcan functions were applied in sequence using default parameters: CAZyme_annotation, gff_process, cgc_finder, and substrate_prediction. A total of 1,096 genes were identified as receiving a GH16_3 annotation in the run_dbcan ‘overview.tsv’ result. The subset of 980 genes with a domain hit from the GH16_3 hmm were trimmed to the identified region, aligned using MAFFT (--globalpair --maxiterate 1000), and manually evaluated for the presence of the characteristic GH16_3 active site (EXDXXE), yielding 972 sequences. Filtering of the sequences to require that they represent only oral associated taxa yielded 543 sequences, with oral-associated defined per the HOMD Genome Taxon Table metadata (April 26, 2026). The final set of 543 oral sequences with a GH16_3 active site were aligned with MAFFT as above. We included two functionally characterized representatives of a closely related and well-supported GH16 subfamily, GH16_8^1^ from the list provided at https://www.cazy.org/GH16_characterized.html:CAR45292.1 (*Streptococcus suis* P1/7) and BAA97991.1 (*Clostridium perfringens* ATCC 10873). To infer phylogenetic relationships we used IQ-Tree^26^ including ModelFinderPlus^27^ to determine the rate substitution model (-m MPF, yielding best-fit model Q.PFAM + F+I+R5, chosen according to BIC), and bootstrap to assess branch support (-B 1000 --bnni --alrt 1000). The tree was visualized using iTOL,^28^ with leaves hyper-linked to allow users to quickly view genomic neighborhoods through the JBrowse functionality provided through HOMD, and annotated with information on taxonomy, presence of predicted signal peptide, and genomic context, see below.

To identify and characterize signal peptides, all 543 oral GH16_3 proteins were analyzed using SignalP6.0^29^ on the SignalP web server (https://services.healthtech.dtu.dk/services/SignalP-6.0/) with predictions based on selection of options for: Organism - “Other”, Output Format - “Long Format”, Model Mode - “Slow”.

To evaluate genomic context around oral GH16_3 proteins we considered: CAZyme Gene Cluster (CGC) and polysaccharide utilization locus (PUL) annotations generated by run_dbcan, Bakta^30^ annotations of GH16_3 neighborhoods (+/− 5 genes around the GH16_3) in the 384 genomes encoding the 543 oral GH16_3 (flags), evaluation of predicted operon structure with the Operon-mapper^31^ webserver (https://biocomputo.ibt.unam.mx/operon_mapper) for select cases, and manual review of genomic neighborhoods using the HOMD JBrowse viewer to evaluate strand coherence and gene proximity.

Conservation of the region around the TfGlcA locus within *T. forsythia* genomes was evaluated using standalone CAGECAT^32^ cblaster v1.4.0 and clinker v0.0.31. Default cblaster searches seeded with the five gene region encompassing the *glcA* operon (*susC-susD-glcA*) together with its upstream ECF regulatory module (*sigG-fecR*) from strain 92A2 were searched against 13 *T. forsythia* genomes (run_dbcan output gene calls) with BLASTp default thresholds (≥ 30% identity, ≥ 50% coverage, e-value ≤ 0.01) to identify genome neighborhoods, clinker was used to visualize neighborhoods.

All GH16_3 gene predictions, with hyperlinks enabling visualization of genomic neighborhoods via HOMD JBrowse tracks are provided in Supplementary Data File 1 along with additional associated taxonomy metadata, annotations, protein sequences, and cross-references to existing HOMD Prokka and NCBI gene identifiers. This dataset further includes computational predictions of N-terminal signal peptides, lipidation sites within encoded enzyme sequences, and predicted β-glucan substrate specificities for each enzyme based on annotation pipelines, access to these detailed outputs is provided, through Zenodo, along with complete run_dbcan analyses of all HOMD genomes (see Data Availability statement).

### AlphaFold2 Structural Prediction and Analysis

Three-dimensional structures of GlcA and GlcB were predicted using AlphaFold2^33^ (local installation or web-based ColabFold implementation, using default settings). Model confidence was evaluated using pLDDT scores and predicted aligned error (PAE) plots. Structural visualization, secondary-structure annotation, and active-site inspection were performed in UCSF ChimeraX^34^. Structural superpositions were generated using the “matchmaker” function to calculate backbone RMSD and assess fold conservation between the two proteins.

## Results

### Structural organization and phylogenetic analysis of T. forsythia β-glucanases.

**Figure 1:**
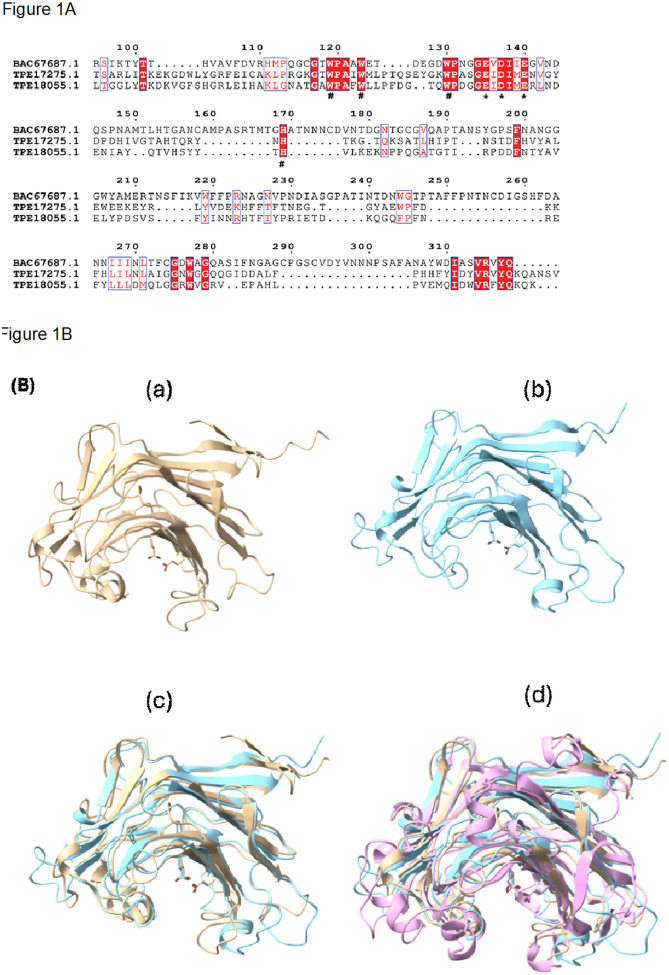
Structural comparison of GlcA and GlcB homologs. A. Multiple sequence alignment of GlcA (TPE17275.1), GlcB (TPE18055.1), and a typical laminarinase (BAC67687.1; Lam 16A from *Phanerochaete chrysosporium*). Catalytic residues forming the GH16 catalytic triad are marked with asterisks (*), and potential active-site residues involved in substrate binding are indicated by hash symbols (#). The figure was prepared using ESPript 3.0 B. AlphaFold2 models of (a) TfGlcA (yellow and (b) TfGlcB (blue). Both enzymes adopt the canonical GH16 jellyroll (β sandwich) fold, consisting of two opposing β-sheets forming a compact β-sandwich. (c & d) TfGlcA and TfGlcB superimposed to each other and to experimentally determined crystal structure (magenta) of Lam 16A (PDB 2W52) from *P. chrysosporium*. T*f*GlcA, superposition with Lam 16A yielded a sequence alignment score of 227.9, with an RMSD of 1.028 Å across 103 pruned Ca atom pairs and RMSD score of 7.703 Å across all 217 pairs. TfGlcB yielded a similarity score of 162.8 with a RMSD score of 1.05 Å across 98 pruned atoms and RMSD of 6.96 across all 222 atom pairs. The overall fold of TfGlcA and TfGlcB closely matched the canonical GH16 β-sandwich architecture, with well-conserved catalytic residues and active-site geometry consistent with the annotated catalytic triad (E, D, E, highlighted as sticks).

A search of the CAZy database showed that *T. forsythia* possesses two GH16 subfamily 3 glucanase homologs, which correspond to GlcA (TPE17275.1) and GlcB (TPE18055.1) in strain ATCC 43037. Pairwise sequence analysis showed that GlcA and GlcB share 31% protein sequence identity and both contain a conserved GH16_3 catalytic motif EXDXXE (residues EIDIME) and active site residues typical of the GH16_3 family (Fig. 1A). AlphaFold2 modeling predicted high-confidence structures for both enzymes, each adopting the canonical jelly-roll β-sandwich fold with a well-defined β-glucan substrate binding cleft containing identically positioned catalytic glutamate residues characteristic of GH16 hydrolases (Fig. 1B). Structural superposition in ChimeraX confirmed extensive conservation between the two proteins, with only minor structural variations. Alignment of TfGlcA and TfGlcB with a typical laminarinase (PDB 2W52) showed that both enzymes share a highly conserved core fold. TfGlcA exhibits higher sequence similarity (score 227.9) and a pruned RMSD score of 1.028 Å over 103 Cα residues. While TfGlcB shows a lower similarity score (162.8), an equally well-preserved structural core with a pruned RMSD of 1.055 Å across 98 atoms. Overall, both glucanases align closely with the laminarinase catalytic architecture, differing mainly in peripheral loop regions. These data confirm that both TfGlcA and TfGlcB belong to GH16 subfamily 3 (GH16_3), which is characterized by a β-jelly roll fold and a conserved catalytic motif (EXDXE).

The SignalP 6.0 server predicted signal peptides of both proteins having lipidation motifs typical of gram-negative bacteria. This suggests that these β-glucanases are lipid-modified and likely associated with the cell envelope, indicating their role in processing of extracellular or cell-associated β-glucans.

**Figure 2:**
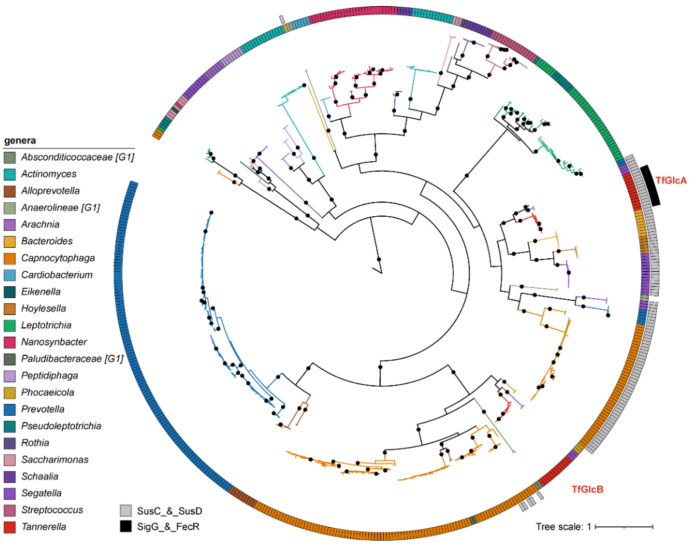
Phylogenetic relationships of oral GH16 subfamily 3 β-glucanases. *Tannerella forsythia*GH16_3 β - glucanases represent two distinct clades, one of which is in sigma/anti-sigma factor regulated polysaccharide utilization cluster (PUL). Tree is based on aligned GH16_3 domains, initial hits in HOMD were filtered to include only those defined as oral-associated and those harboring the characteristic active site (EXDXXE); two GH16_8 sequences were included as outgroups (see Methods for details). Bootstrap support of SH-aLRT ≥80% and UFboot ≥95% indicated with filled black circle. Colored strips indicate genus, grey strips indicate presence of SusC and SusD transporter components, black strips indicate presence of sigma factor SigG and anti-sigma factor FecR. An interactive version of the tree can be found online https://itol.embl.de/tree/70177211213229331779828815 and shows full protein IDs and the ability to follow hyperlink to JBrowse views of genomic neighborhoods on the HOMD webserver. Underlying data is available in Supplementary Data File 1 and on Zenodo.

A search of all genomes in the HOMD database identified GH16_3 β-glucanase genes in 10% of oral species (543 total sequences), representing phylogenetically diverse taxa (Fig. 2). The majority of GH16_3 were identified in Bacteroidota (361 sequences in 26 species), Actinomycetota (61 sequences in 9 species), Fusobacteriota (50 sequences in 8 species), episymbiont Candidate Phyla Radiation Patescibacteria (43 sequences in 5 species), and the Bacillota (17 sequences in 2 species), as well as others (Fig. 2).

**Figure 3:**
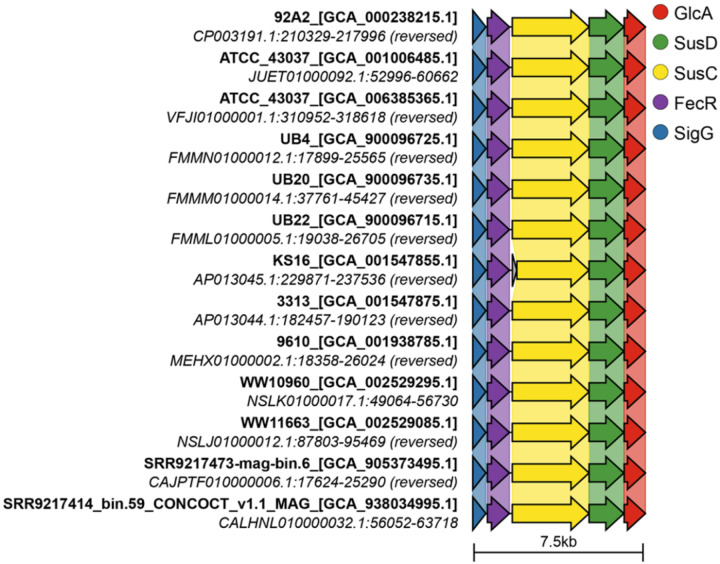
Conservation of the TfGlcA locus. The analysis shows that the entire genetic region encompassing the *glcA*operon (*susC-susD-glcA*), together with its upstream ECF regulatory module (*sigG-fecR*), is highly conserved across all sequenced *T. forsythia* strains. Conservation of operon architecture was assessed using CAGECAT's cluster comparison workflow (standalone cblaster and clinker, see Methods). Colored blocks indicate different proteins and colored shading indicates ≥90% protein sequence identify.

Phylogenetic analysis showed that *Tannerella* GH16_3 hydrolases, TfGlcA and TfGlcB, segregate into two separate clades. CAZyme Gene Cluster and Polysaccharide Utilization Locus annotations generated by run_dbcan, supported by Bakta annotations of GH16_3 neighborhoods indicated that TfGlcA is embedded within a conserved locus containing SusC/SusD-like transporter genes and located immediately downstream of an extracytoplasmic function (ECF) regulatory module comprising of an ECF sigma factor, TfSigG, and its anti-sigma factor, TfFecR (Fig. 3). This is consistent with our previous demonstration that this ECF regulatory module controls the expression of TfGlcA expression in response to *F. nucleatum*^15,16^. CAGECAT (CompArative GEne Cluster Analysis Toolbox) analysis revealed that the entire genetic locus comprising the glcA operon along with the ECF regulators is highly conserved among all sequenced *T. forsythia* strains, with no evidence of major rearrangement or loss (Fig. 3). The genomic neighborhood of the TfGlcA genes is typical of inducible polysaccharide uptake loci (PULs) in gut Bacteroidetes, and this clade appears most closely related to GH16_3 sequences in PUL-like loci in *Prevotella* and *Capnocytophaga*, which however lack the adjacent sigma/anti-sigma regulatory module. The specific biological roles and regulatory mechanisms governing these GH16_3 glucanases remain to be determined. In contrast, TfGlcB sits within a distinct clade of β-glucanases that do not share the PUL-like architecture and are encoded by diverse species in the Bacteroidota.

### Expression and purification of recombinant T. forsythia GlcA and GlcB

**Figure 4:**
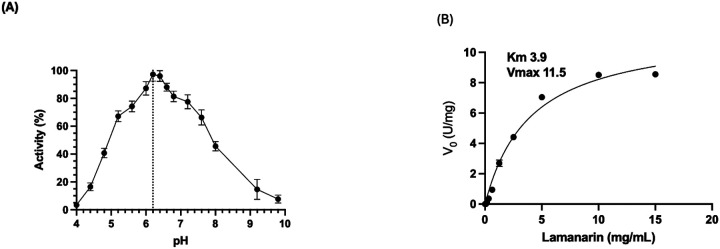
pH optimum and substrate-dependent kinetics of TfGlcA. A. Enzyme activity of TfGlcA at different pH values. Data represent means ± S.D of three independent experiments, with duplicate technical measurements performed for each pH condition. B. Michaelis–Menten plot showing the effect of laminarin (substrate) concentration on TfGlcA activity. K_m_ and V_max_ were determined by fitting the data to the Michaelis-Menten equation.

The TfGlcA protein, engineered with an N-terminal GST (glutathione S-transferase) and a C-terminal 6xHis tag, was purified from *E. coli* lysates using Ni affinity chromatography. The resulting 63 kDa fusion protein exhibited enzymatic activity toward lichenin β-glucan, as demonstrated by zymography (Fig. 4). Following thrombin digestion, the protein product was subjected to a second round of affinity chromatography, yielding a highly purified rTfGlcA active enzyme preparation that appeared as a single band on SDS-PAGE at the expected size (32 kDa) (Fig. 4).

In contrast, TfGlcB could not be obtained in an active form despite multiple optimization attempts, including low temperature expression and the use of the Rosetta *E. coli* strain to enhance codon compatibility and protein solubility. TfGlcB protein expressed exclusively in the insoluble fraction localized to inclusion bodies. We attempted to purify the insoluble protein from the inclusion bodies with 8M urea solubilization and Ni-chromatography, followed by refolding the protein through stepwise (6M, 4M, 2M 1M urea and finally TBS alone) dialysis. In some experiments glycerol was included during dialysis to promote refolding. Despite these refolding attempts, the recovered protein (Figure S2) showed no detectable activity toward lichenin by zymography or toward laminarin in the DNS assay (data not shown).

### pH and Temperature Optima and Substrate Specificity of TfGlcA

**Figure 5:**
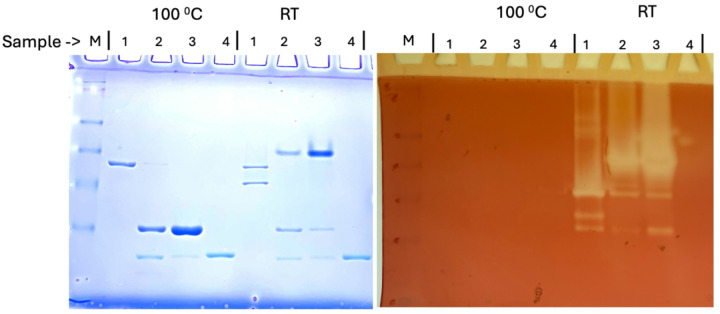
Analysis of expression and purification of recombinant TfGlcA enzyme. A. SDS-PAGE analysis of TfGlcA purification and tag removal. Samples: Lane M, prestained protein markers; lane 1, purified GST–GlcA6xHis fusion protein; lane 2, thrombin digest of GST–GlcA6xHis protein; lane 3, Ni-bound fraction (GlcA) of Thrombin digest; lane 4, Ni-unbound fraction (GST) of thrombin digest. Samples were either heat-denatured at 100 ^0^C for 3 min or applied directly before electrophoresis. B. Zymogram analysis using lichenin β-glucan as substrate. Clear bands against the stained background indicate enzymatic activity,

TfGlcA showed optimal activity at 40°C (data not shown) and at a pH of 6.2 when laminarin was used as the substrate (Fig. 5A). These optimized reaction conditions were applied for subsequent substrate specificity assays. Substrate preferences of TfGlcA were determined by measuring enzymatic activity against a panel of polysaccharides differing in monosaccharide composition and linkage patterns (Table 1). TfGlcA displayed the highest activity toward laminarin and lichenin, with relative activities of 100% (4.4 U/mg) and 82% (3.60 U/mg), respectively. Kinetic parameters were determined by fitting the laminarin dependent reaction velocities to the Michaelis-Menten model using Prism software. TfGlcA exhibited a *K*_*m*_ of 10.28 mg/ml and a *V*_*max*_ of 13.34 U/mg for laminarin hydrolysis (Fig. 5B).

## Discussion

In this study we demonstrated that TfGlcA can digest mixed linkage β-glucans and cleave β(1,3) glycosidic linkages. The substrate specificity profile of TfGlcA β-glucanase demonstrated it exhibits highest catalytic activity toward laminarin, followed by lichenin (88% relative activity), while showing substantially lower activity toward yeast β-glucan (23%) and no detectable activity toward CMC or chitin. This pattern is consistent with enzymes that preferentially act on β(1,3) linked glucans with mixed β(1,3/1,6)- branching. The distinctly higher activity toward laminarin, a primarily β(1,3) glucan with β(1,6) branches, suggests that Tf GlcA likely possesses an active site-cleft optimized for accommodating β(1,3) linkages while tolerating limited branching. The strong activity against lichenin, having both β(1,3 and 1,4) linkages indicates that the enzyme can also hydrolyze substrates with mixed linkage structures with slightly reduced efficiency compared to laminarin. The reduced activity toward yeast glucan plausibly is due to highly branched structure of the β-glucan with dense β(1,6)- linkages, which may sterically hinder β-glucan accessibility and productive binding to the enzyme. The lack of activity against CMC, a β1,4 cellulose derivative, supports the conclusion that *T. forsythia* GlcA is not a classical endo β(1,4) glucanase and lacks catalytic features for cleaving β(1,4)- linkages. The complete lack of activity toward chitin, a β(1,4) linked N-acetylglucosamine polymer, shows that TfGlcA does not act on acetylated hexosamine substrates and is therefore not a chitinolytic enzyme. Together, these data indicate that *T. forsythia* GlcA is a β(1,3) glucanase with capability to cleave mixed β(1,3/1,4) substrates and limited tolerance for dense branching. This substrate preference is consistent with β(1,3) glucanase/laminarinase. A relatively high *K*_*m*_ (3.9 mg/mL) for laminarin suggests that TfGlcA has low substrate affinity and is adapted to function under conditions where β-glucans are relatively abundant, such as from dietary β-glucans in the oral niche following dietary intake. The *glcA* operon, along with the associated ECF regulatory module, is highly conserved in sequenced strains of *T. forsythia*. This conservation suggests selective pressure to maintain TfGlcA activity in periodontitis pathogenesis, as well as its regulation within the species. Interestingly, the health-associated *Tannerella* species *T. serpentiformis* is predicted to lack β-glucanase orthologs.

The combined sequence, structural, and phylogenetic analyses indicate that *T. forsythia* encodes two evolutionary distinct GH16_3 β-glucanases that might be involved in β-glucan metabolism. However, the enzymatic specificity of TfGlcB could not be determined since the GlcB protein could not be expressed recombinantly in *E. coli* in an active form. The strong structural conservation between GlcA and GlcB - including the preserved jelly roll fold and identical placement of catalytic residues- suggests a shared catalytic mechanism, and subtle loop differences near the substrate binding cleft reflect preference toward distinct glucan substrates. Phylogenetic placement of GH16 _3 hydrolases in *Tannerella* within two separate clades highlights functional divergence whereby TfGlcA resides within a polysaccharide uptake locus (PUL) regulated by an ECF sigma/anti-sigma (TfSigG/TfFecR) system. This is consistent with an inducible glucan acquisition module responsive to environmental cues such as *F. nucleatum*^16^. In contrast, TfGlcB sits in a distinct clade of GH16_3 enzymes lacking the PUL-like architecture and regulation, suggesting that it represents a more generalist glucanase. Together, these findings support a model in which *T. forsythia* employs both a specialized, regulated PUL associated glucanase (TfglcA) and a conventional GH16 enzyme (GlcB) to adapt to the dynamic polysaccharide landscape of the oral niche, rich in diverse dietary β-glucans. Dietary β-glucans occur in multiple structural forms: mixed linkage β(1,3/1,4) glucans (MLGs) found in cereals such as oats and barley, β(1,3)linked callose in plant cell walls, and β(1,3)(1,6) branched glucans present in edible fungi and seaweeds^17,18^. Given that dietary intake provides a wide variety of β-glucan polysaccharides, it is plausible that TfGlcA as well as other yet to be characterized oral bacterial β-glucanases contribute to the degradation of these complex carbohydrates in the oral environment.

Conceivably, *Candida albicans*, a fungal commensal organism of the oral microbiome, represents an additional potential source of β-glucan substrate for *T. forsythia*. Although we did not observe any antifungal activity of TfGlcA against *C. albicans* (data not shown), the enzyme may be involved in liberating glucose oligomers from the yeast cell wall. There is strong evidence that *C. albicans* releases and exposes β−1,3-glucan in the oral environment, particularly within biofilm matrices^19,20^. It would be interesting to determine whether *T. forsythia* and *F. nucleatum* are increasingly found with *Candida* in mixed biofilms. Nevertheless, TfGlcA, through hydrolysis of β-glucans could release short chain glucose oligosaccharides that serve as carbon sources for ATP generation by coexisting saccharolytic oral bacteria, including *F. nucleatum*, as we have demonstrated previously^15^. In contrast, *T. forsythia* is asaccharolytic and therefore does not benefit directly from the released glucose oligosaccharides. Intriguingly, *T. forsythia* has the unique ability to produce copious amounts of methylglyoxal (MGO), a dicarbonyl toxic compound, from dihydroxyacetone phosphate (DHAP) by methylglyoxal synthase enzyme (MgsA) during glucose fermentation^35^. Thus, *T. forsythia* can utilize glucose and short-chain glucose oligosaccharides released from β-glucan degradation to produce MGO. We find that digestion of laminarin by TfGlcA generates free glucose in addition to glucose oligosaccharides, as judged by thin layer chromatography analysis (data not shown). It is also likely that glucose oligosaccharides entering the cell via Sus transporters are further degraded by cellular glycosidases to release free glucose, that then becomes available for MGO generation. The impact of β-glucan degradation by TfGlcA could directly impact both the microbial ecology and the host. While MGO may exert cytotoxic stress on *T. forsythia* and other species, MGO promotes the formation of inflammogenic advanced glycation end products (AGEs) that contribute to periodontal inflammation and tissue destruction^36^. This, in turn, increases the availability of host derived peptides and heme as key nutrient sources for the growth of asaccharolytic anaerobes, including *T. forsythia*. These findings support a role for β-glucan utilization via β-glucanases in shaping the oral microbial ecology and inflammatory processes.

### Supplementary Files

This is a list of supplementary files associated with this preprint. Click to download.

- FINAL.MANUSCRIPT.HOMDGH163.v2026.150.1214.xlsx
- SupplementaryData.docx

## Figures and Tables

**Table 1:** TfGlcA activity against different polysaccharides

Substrate	Linkages	Relative Activity, %
Lamanarin	[Glcb1–3Glcb1–3(Glcb1–6)Glcb1–3 Glcb1–3]_n_	100^a^
Lichenin	[Glcb1–4Glcb1–3Glcb1–4]_n_	82^b^
Barley β-glucan	[Glcb1–4Glcb1–4Glcb1–3Glcb1–4]_n_	65^c^
Yeast β-glucan	[Glcb1–3Glcb1–3(Glcb1–6)Glcb1–3 Glcb1–3Glcb1–3]_n_	28^d^
CMC	[Glcb1–4 Glcb1–4]_n_	0
Chitin	[GlcNAcb1–4 GlcNAcb1–4]_n_	0

The different letters (^a^, ^b^, ^c^ and ^d^) indicate significant differences (*p* < 0.05).

## Data Availability

In addition to the summary data provided in Supplementary Data File 1, additional data associated with described bioinformatic analyses are available at https://zenodo.org/records/20358499 (doi:10.5281/zenodo.20358499), including: results of the run_dbcan analysis of 8,177 HOMD v11.02 genomes (representing protein calls distinct from those currently available on HOMD); protein sequences, alignments, and tree files; Bakta annotations of the 384 genomes identified as encoding the 543 GH16_3 sequences in the tree; and outputs of operon-mapper and signalp6.0 webserver analyses.

## References

[1] Tanner A. C. & Izard J. *Tannerella forsythia*, a periodontal pathogen entering the genomic era. Periodontol. 2000 42, 88–113 (2006). 10.1111/j.1600-0757.2006.00184.x 16930308

[2] Sharma A. Virulence mechanisms of *Tannerella forsythia*. Periodontol. 2000 54, 106–116 (2010). 10.1111/j.1600-0757.2009.00332.x PMC2934765 20712636

[3] Pu R. Association between oral microbial diversity and periodontitis in a nationally representative U.S. population: A cross-sectional study. J. Periodontol. (2026). 10.1002/jper.70101 PMC13380331 42210032

[4] Sigusch B. Peridontopathogenic key species in correlation to the current classification system. Clin. Oral Investig. 29, 339 (2025). 10.1007/s00784-025-06413-2 PMC12159085 40500387

[5] Werner N. The association between periodontal microbial biomarkers and primary therapy outcome. Clin. Oral Investig. 28, 523 (2024). 10.1007/s00784-024-05904-y PMC11399289 39269543

[6] Chen T., Marsh P. D. & Al-Hebshi N. N. SMDI: An Index for Measuring Subgingival Microbial Dysbiosis. J. Dent. Res. 101, 331–338 (2022). 10.1177/00220345211035775 34428955 PMC8982011

[7] Ribeiro A. C., Matarazzo F., Faveri M., Zezell D. M. & Mayer M. P. Exploring Bacterial Diversity of Endodontic Microbiota by Cloning and Sequencing 16S rRNA. J. Endod. 37, 922–926 (2011). 10.1016/j.joen.2011.04.007 21689545

[8] Rocas I. N., Alves F. R., Santos A. L., Rosado A. S. & Siqueira J. F. Jr. Apical Root Canal Microbiota as Determined by Reverse-capture Checkerboard Analysis of Cryogenically Ground Root Samples from Teeth with Apical Periodontitis. J. Endod. 36, 1617–1621 (2010). 10.1016/j.joen.2010.07.001 20850664

[9] Espinoza-Arrue J. Profiling the Bacterial Microbiome Across Peri-Implant Conditions. J. Clin. Periodontol. (2025). 10.1111/jcpe.70024 PMC13402070 40858525

[10] Dantas L. O. Metatranscriptomic Insights into Bacterial Activity, Virulence, and Antimicrobial Resistance in the Root Canal Microbiome of Acute Apical Abscesses. J. Endod. (2026). 10.1016/j.joen.2026.01.002 41571088

[11] Sharma A., Inagaki S., Sigurdson W. & Kuramitsu H. K. Synergy between *Tannerella forsythia* and *Fusobacterium nucleatum* in biofilm formation. Oral Microbiol. Immunol. 20, 39–42 (2005). 15612944 10.1111/j.1399-302X.2004.00175.x

[12] Zijnge V., Ammann T., Thurnheer T. & Gmür R. in Subgingival biofilm structure (eds Mombelli A. & Kinane D. F.) (Karger, 2012). 10.1159/000329667 22142954

[13] Zijnge V. Oral biofilm architecture on natural teeth. PloS one 5, e9321 (2010). 10.1371/journal.pone.0009321 20195365 PMC2827546

[14] Ammann T. W., Gmür R. & Thurnheer T. Advancement of the 10-species subgingival Zurich Biofilm model by examining different nutritional conditions and defining the structure of the in vitrobiofilms. BMC microbiology 12, 227 (2012). 10.1186/1471-2180-12-227 23040057 PMC3561252

[15] Honma K., Ruscitto A. & Sharma A. beta-Glucanase Activity of the Oral Bacterium Tannerella forsythia Contributes to the Growth of a Partner Species, Fusobacterium nucleatum, in Cobiofilms. Appl. Environ. Microbiol. 84 (2018). 10.1128/AEM.01759-17 PMC5734031 29079615

[16] Honma K., Sasaki H., Hamada N. & Sharma A. An Extracytoplasmic Function Sigma/Anti-Sigma Factor System Regulates beta-Glucanase Expression in Tannerella forsythia in Response to Fusobacterium nucleatum Sensing. J. Bacteriol., e0031322 (2022). 10.1128/jb.00313-22 36448787 PMC9765289

[17] Chang S. C., Saldivar R. K., Liang P. H. & Hsieh Y. S. Y. Structures, Biosynthesis, and Physiological Functions of (1,3;1,4)-beta-D-Glucans. Cells 10 (2021). 10.3390/cells10030510 PMC7997180 33673640

[18] Du B., Meenu M., Liu H. & Xu B. A Concise Review on the Molecular Structure and Function Relationship of beta-Glucan. Int J Mol Sci 20 (2019). 10.3390/ijms20164032 PMC6720260 31426608

[19] Chen T., Wagner A. S. & Reynolds T. B. When Is It Appropriate to Take Off the Mask? Signaling Pathways That Regulate ss(1,3)-Glucan Exposure in Candida albicans. Front Fungal Biol 3 (2022). 10.3389/ffunb.2022.842501 PMC10003681 36908584

[20] Taff H. T. A Candida biofilm-induced pathway for matrix glucan delivery: implications for drug resistance. PLoS Pathog 8, e1002848 (2012). 10.1371/journal.ppat.1002848 22876186 PMC3410897

[21] Yu N. Y. PSORTb 3.0: improved protein subcellular localization prediction with refined localization subcategories and predictive capabilities for all prokaryotes. Bioinformatics 26, 1608–1615 (2010). 10.1093/bioinformatics/btq249 20472543 PMC2887053

[22] Li H. & McKee L. S. Measuring Enzyme Kinetics of Glycoside Hydrolases Using the 3,5-Dinitrosalicylic Acid Assay. 10.1007/978-1-0716-3151-5_2 37149520

[23] Chen T. The Human Oral Microbiome Database: a web accessible resource for investigating oral microbe taxonomic and genomic information. Database (Oxford) 2010, baq013 (2010). 10.1093/database/baq013 PMC2911848 20624719

[24] Escapa I. F. New Insights into Human Nostril Microbiome from the Expanded Human Oral Microbiome Database (eHOMD): a Resource for the Microbiome of the Human Aerodigestive Tract. mSystems 3 (2018). 10.1128/mSystems.00187-18 PMC6280432 30534599

[25] Zheng J. Carbohydrate-active enzyme annotation in microbiomes using dbCAN. BioRxiv (2024). 10.1101/2024.01.10.575125

[26] Wong T. K. F. IQ-TREE 3: phylogenomic inference software using complex evolutionary models. Mol. Biol. Evol. 43 (2026). 10.1093/molbev/msag117 PMC13191116 42085559

[27] Kalyaanamoorthy S., Minh B. Q., Wong T. K. F., von Haeseler A. & Jermiin L. S. ModelFinder: fast model selection for accurate phylogenetic estimates. Nat Methods 14, 587–589 (2017). 10.1038/nmeth.4285 28481363 PMC5453245

[28] Letunic I. & Bork P. Interactive Tree of Life (iTOL) v6: recent updates to the phylogenetic tree display and annotation tool. Nucleic Acids Res 52, W78–W82 (2024). 10.1093/nar/gkae268 38613393 PMC11223838

[29] Teufel F. SignalP 6.0 predicts all five types of signal peptides using protein language models. Nat. Biotechnol. 40, 1023–1025 (2022). 10.1038/s41587-021-01156-3 34980915 PMC9287161

[30] Schwengers O. Bakta: rapid and standardized annotation of bacterial genomes via alignment-free sequence identification. Microb Genom 7 (2021). 10.1099/mgen.0.000685 PMC8743544 34739369

[31] Taboada B., Estrada K., Ciria R. & Merino E. Operon-mapper: a web server for precise operon identification in bacterial and archaeal genomes. Bioinformatics 34, 4118–4120 (2018). 10.1093/bioinformatics/bty496 29931111 PMC6247939

[32] van den Belt M. CAGECAT: The CompArative GEne Cluster Analysis Toolbox for rapid search and visualisation of homologous gene clusters. BMC Bioinformatics 24, 181 (2023). 10.1186/s12859-023-05311-2 37131131 PMC10155394

[33] Jumper J. Highly accurate protein structure prediction with AlphaFold. Nature 596, 583–589 (2021). 10.1038/s41586-021-03819-2 34265844 PMC8371605

[34] Meng E. C. UCSF ChimeraX: Tools for structure building and analysis. Protein Sci. 32, e4792 (2023). 10.1002/pro.4792 37774136 PMC10588335

[35] Settem R. P. Tannerella forsythia produced methylglyoxal causes advanced glycation endproducts (AGEs) accumulation to trigger cytokine secretion in human monocytes. Mol Oral Microbiol 33, 292–299 (2018). 10.1111/omi.12224 29573211 PMC6041129

[36] Settem R. P. & Sharma A. Oral bacterium contributes to periodontal inflammation by forming advanced glycation end products. Infect. Immun. 93, e0056024 (2025). 10.1128/iai.00560-24 40172539 PMC12070732

